# Unexpected outcome after sclerotherapy of simple renal cyst

**DOI:** 10.1186/1471-2369-13-63

**Published:** 2012-07-24

**Authors:** Yasuhiro Hashimoto, Atsushi Imai, Noriko Tokui, Atsushi Sasaki, Hisao Saitoh, Takuya Koie, Chikara Ohyama

**Affiliations:** 1 Oyokyo Kidney Research Institute Hirosaki Hospital, 90 Yamazaki Kozawa, Hirosaki, Aomori 036-8243, Japan; 2 Department of Urology, Hirosaki University Graduate School of Medicine, 5 Zaifucho, Hirosaki, Aomori 036-8562, Japan

**Keywords:** Papillary renal cell carcinoma, Simple renal cyst, Sclerotherapy, Malignant transformation

## Abstract

**Background:**

Simple renal cysts usually have benign clinical features. We report a rare case of papillary renal cell carcinoma (RCC) associated with a large recurrent simple cyst following sclerotherapy.

**Case Presentation:**

A 47-year-old Japanese woman received minocycline sclerotherapy for a large (9 cm in diameter) simple left renal cyst in May 2005. The cyst regrew, and second-line sclerotherapy with ethanol was performed in November 2005. Three years later, she developed papillary RCC on the wall of the recurrent renal cyst. Radical nephrectomy was performed, but the patient died of metastatic disease 15 months after surgery.

**Conclusion:**

Malignant transformation from recurrent simple renal cyst to RCC may occur in the years following sclerotherapy, underscoring the need for long-term follow-up.

## Background

Simple renal cysts are common, particularly in the elderly. Indeed, 50 % of individuals over the age of 50 years exhibit single or multiple renal cysts [1]. While most renal cysts are benign and left untreated, large or symptomatic cysts must be excised or destroyed. Renal cyst aspiration, sclerotherapy, and laparoscopic removal are safe and effective treatments for symptomatic simple renal cysts [2][3][4][5].

Only two published reports have documenting the malignant transformation of a simple renal cyst into renal cell carcinoma [6][7], and no case study published in English has described malignant transformation of a renal cyst following sclerotherapy. We report a rare case of papillary renal cell carcinoma that developed on the wall of a large recurrent renal cyst three years following sclerotherapy.

## Case presentation

**Figure 1:**
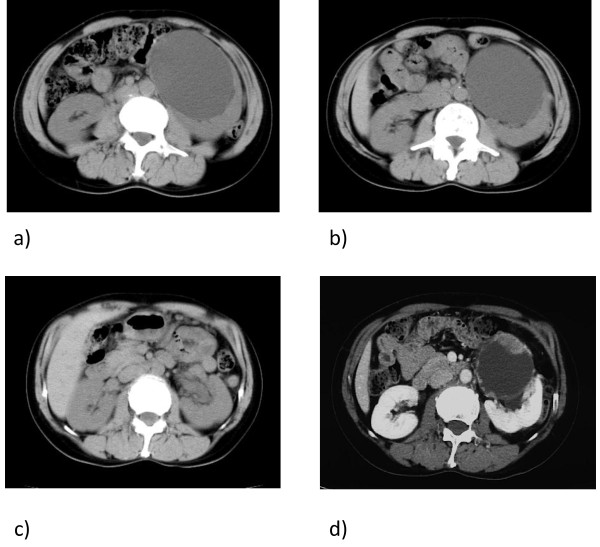
**(a) Abdominal CT acquired in May 2005.CT images revealed a renal cyst approximately 9 cm in diameter in the middle of the left kidney.** Renal cyst sclerotherapy with minocycline was performed in May 2005. **(b)** Abdominal CT acquired in November 2005. After sclerotherapy with minocycline, the renal cyst regrew to its original size within 6 months. Therefore, sclerotherapy with 95 % ethanol was administered. **(c)** Abdominal CT from July 2007. The CT images showed no regrowth of the renal cyst nearly two years after second-line ethanol sclerotherapy. **(d)** Enhanced abdominal CT from January 2009 revealed a cystic renal mass approximately 7 cm in diameter containing an irregular enhanced mass lesion.

A 47-year-old Japanese woman received sclerotherapy for a large simple left renal cyst in May 2005 (Figure 1a). Following aspiration of cyst fluid (approximately 300 ml), we injected 200 mg minocycline into the cyst cavity. The cyst regrew, however, so a single 20-min sclerotherapy procedure using 95 % ethanol was performed in November 2005[5] (Figure 1b). The aspirated fluid obtained during the second procedure was clear yellow and cytological examination showed no signs of malignancy. For two years after second-line sclerotherapy, follow-up examination revealed no signs of cyst regrowth (Figure 1c).

**Figure 2:**
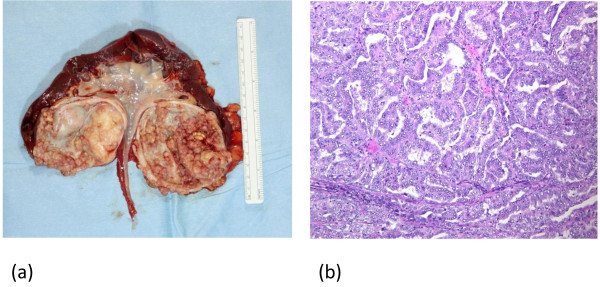
**(a) Macroscopic view of the surgical specimen.** A cystic renal tumor was found in the middle of the left kidney that was unconnected to the pelvis renalis. The tumor arising from the cystic wall was gray and multinodular. **(b)** Microscopic examination revealed that tumor cells with high atypia exhibited tubular and papillary growth patterns (original magnification × 100).

In December 2008, the patient experienced gross hematuria and was referred to our hospital in January 2009. Computed tomography revealed a tumor emerging from the inner wall of the recurrent renal cyst (Figure 1d). However, there was yet no evidence of metastasis. Left radical nephrectomy was performed under the diagnosis of cystic renal cell carcinoma in February 2009. Gross examination of the excised tissue revealed numerous fragile and easily detachable tumors on the inner cyst wall(Figure 2a). The tumors penetrated to the outer cyst wall and had already invaded the section of visceral peritoneum excised during radical nephrectomy. There were no indications of peritonitis carcinomatosa.

Microscopic examination of the cyst revealed relatively large tumor cells with abundant eosinophilic cytoplasm. The nuclei were enlarged, with prominent eosinophilic nucleoli, and most were vesicular. The cells exhibited medium to high cytological atypia. Tumors exhibiting papillary and tubular growth patterns were observed (Figure 2b). Microscopic examination of the excised kidney indicated that malignant cells from the cyst had invaded the renal parenchyma. Immunohistochemical analysis showed that tumor cells were RCC(−), AMACR(+), 34βE12(−), CK7(+), and either CD10(+) or CD10(−). The pathological diagnosis was type II papillary renal cell carcinoma.

At the time of radical nephrectomy, metastasis was not detected in the resected para-aortic lymph nodes. In April 2009, however, multiple lung metastases were found by follow-up CT examination. The patient received sunitinib (37.5 mg/day) followed by sorafenib (800 mg/day), but her disease progressed. Irradiation at a dose of 30 Gy was administered to a metastatic bone lesion at Th7–Th11 in December 2009. Despite treatment, the patient died of metastatic disease in May 2010.

## Discussion

Simple renal cysts are usually benign and easily detected by routine sonography if the following criteria are met: (i) absence of internal echoes; (ii) the presence of a sharply defined, thin, distinct wall with a smooth and distinct margin; (iii) adequate sound wave transmission through the cyst with acoustic enhancement behind it; and (iv) a spherical or slightly ovoid shape[8]. In the case presented, sonography and CT findings led to a diagnosis of simple renal cyst. Cytological examination of the cyst fluid revealed no signs of malignancy and the cyst did not regrow for several years following ethanol sclerotherapy. However, renal cell carcinoma was detected within the recurrent cyst three years later, suggesting a rare occurrence of malignant transformation.

Sasaki *et al.* reported one of the few cases of RCC derived from a simple renal cyst. In their patient, the transition was followed by ultrasonography over six years. Left radical nephrectomy was performed and pathologic examination confirmed the diagnosis of a large (6 cm in diameter) RCC [6]. Cheng-Jui Lin *et al.* reported the case of a huge (11.5 × 9 × 16 cm)simple renal cyst that regrew soon after aspiration, necessitating nephrectomy three months later to relieve intractable flank pain. Again, pathological examination confirmed papillary RCC. [7]

We searched PubMed using the terms“renal cyst”, “carcinoma”, and “sclerotherapy”, but could not find a single case of malignant transformation of a simple renal cyst following sclerotheraphy. There are sporadic case reports of esophageal cancer developing months to years following sclerotherapy to treat esophageal varices. [9][10][11]However, no clear cause-effect relationship has been established, primarily because these patients were treated using a variety of sclerosants.

Our case was similar to that reported by Cheng-Jui Lin *et al.*[7]. In our case, a large simple renal cyst regrew rapidly after first-line minocycline sclerotherapy and again, with a longer delay, after second-line ethanol sclerotherapy. The growth of tumors on the inner cyst wall suggested malignant transformation, but it remains uncertain if sclerotherapy increases the risk of tumorigenesis. Nonetheless, based on this report and those cited, malignant transformation should be considered a possible danger if a large simple renal cyst regrows after renal aspiration or sclerotherapy. Based on the long delay between the last sclerotherapy procedure and the diagnosis of RCC, we recommend long-term follow-up with CT and sonography at least every six months for patients exhibiting recurrent large renal cysts.

## Conclusion

We report a rare case of papillary renal cell carcinoma arising within a recurrent simple renal cyst following sclerotherapy. Unfortunately, the patient died one year and three months after surgery. This case report should alert physicians to the possibility of malignant transformation of simple renal cysts even years after sclerotherapy.

## Competing interests

The authors declare that they have no competing interest.

## Author contributions

All authors participated in the patient’s medical treatment. YH drafted the first version of the manuscript and CO helped to draft the revised manuscript. All authors have read and approved of this submission.

## Pre-publication history

The pre-publication history for this paper can be accessed here:

http://www.biomedcentral.com/1471-2369/13/63/prepub
